# Mapping Glucose Uptake, Transport and Metabolism in the Bovine Lens Cortex

**DOI:** 10.3389/fphys.2022.901407

**Published:** 2022-05-31

**Authors:** Ali Zahraei, George Guo, Kyriakos G. Varnava, Nicholas J. Demarais, Paul J. Donaldson, Angus C. Grey

**Affiliations:** ^1^ Department of Physiology in the School of Medical Sciences, Auckland, New Zealand; ^2^ Mass Spectrometry Hub, Auckland, New Zealand; ^3^ School of Biological Sciences, University of Auckland, Auckland, New Zealand

**Keywords:** lens, metabolites, glucose, MALDI imaging mass spectrometry (IMS), glucose transporter

## Abstract

**Purpose:** To spatially correlate the pattern of glucose uptake to glucose transporter distributions in cultured lenses and map glucose metabolism in different lens regions.

**Methods:** *Ex vivo* bovine lenses were incubated in artificial aqueous humour containing normoglycaemic stable isotopically-labelled (SIL) glucose (5 mM) for 5 min-20 h. Following incubations, lenses were frozen for subsequent matrix-assisted laser desorption/ionisation (MALDI) imaging mass spectrometry (IMS) analysis using high resolution mass spectrometry. Manually dissected, SIL-incubated lenses were subjected to gas chromatography-mass spectrometry (GC-MS) to verify the identity of metabolites detected by MALDI-IMS. Normal, unincubated lenses were manually dissected into epithelium flat mounts and fibre cell fractions and then subjected to either gel-based proteomic analysis (Gel-LC/MS) to detect facilitative glucose transporters (GLUTs) by liquid chromatography tandem mass spectrometry (LC-MS/MS). Indirect immunofluorescence and confocal microscopy of axial lens sections from unincubated fixed lenses labelled with primary antibodies specific for GLUT 1 or GLUT 3 were utilised for protein localisation.

**Results:** SIL glucose uptake at 5 min was concentrated in the equatorial region of the lens. At later timepoints, glucose gradually distributed throughout the epithelium and the cortical lens fibres, and eventually the deeper lens nucleus. SIL glucose metabolites found in glycolysis, the sorbitol pathway, the pentose phosphate pathway, and UDP-glucose formation were mapped to specific lens regions, with distinct regional signal changes up to 20 h of incubation. Spatial proteomic analysis of the lens epithelium detected GLUT1 and GLUT3. GLUT3 was in higher abundance than GLUT1 throughout the epithelium, while GLUT1 was more abundant in lens fibre cells. Immunohistochemical mapping localised GLUT1 to epithelial and cortical fibre cell membranes.

**Conclusion:** The major uptake site of glucose in the bovine lens has been mapped to the lens equator. SIL glucose is rapidly metabolised in epithelial and fibre cells to many metabolites, which are most abundant in the metabolically more active cortical fibre cells in comparison to central fibres, with low levels of metabolic activity observed in the nucleus.

## Introduction

As an avascular, transparent organ in the anterior eye, the ocular lens plays a crucial role in our sense of sight (Donaldson et al., 2017). The transparent and refractive properties of the lens are established by a unique tissue architecture that minimises light scattering and a geometry and refractive index gradient (GRIN) that contributes to the correct focussing of light onto the retina (Donaldson et al., 2017). The anterior surface of the lens consists of a single layer of epithelial cells which at the lens equator divide and initiate a process of cell differentiation to form highly elongated secondary lens fibre cells that reside in the outer cortex of the lens. As part of this differentiation process, cell nuclei and other organelles such as mitochondria are degraded in these fibre cells in order to remove these light scattering elements from the light pathway (Bassnett, 2002; Bassnett, 2009). In addition, these differentiating fibre (DF) cells express new proteins, including the crystallins, which contribute to the formation of the GRIN (Uhlhorn et al., 2008). When DF cells lose their organelles, they become mature fibre cells in the inner cortex of the lens that in turn surround the primary fibre cells that were initially laid down *in utero* and which form the nucleus or core of the lens (McAvoy et al., 1999). This continual process of epithelial cell division, fibre cell differentiation and internalization of existing cells means that the lens grows throughout life and that a gradient of fibre cells of different ages with distinctly different properties and metabolic demands exists in the lens. While it is the structural organisation of the lens that establishes the transparent and refractive properties of the lens, the lens is not a passive optical element (Donaldson et al., 2017), but a biological tissue that requires the input of energy to drive the structural and functional processes that actively maintain its optical properties. The primary source of energy for the lens is glucose, which in the absence of a blood supply must be directly sourced from the surrounding ocular humours, delivered to the different regions of the lens and subsequently metabolised.

In lieu of a blood supply, glucose is taken up from its surrounding humours, where it is present in the aqueous and vitreous humour at approximately 3.2 mM (Davies et al., 1984) and 3.0 mM (Kokavec et al., 2016) in humans, respectively. While the normal concentration of glucose within the lens itself varies between species, it is in the order of 10 mg/100 g tissue (Kuck, 1965). The mechanism of how glucose is delivered and taken up from the surrounding humours has attracted significant research, and our understanding of these mechanisms has developed over time. It was initially hypothesized that glucose was only delivered to and taken up by the epithelial cells located on the lens anterior surface, with the transfer of glucose to the underlying fibre cell mass occurring via passive diffusion via a gap junction-mediated pathway (Goodenough et al., 1980). However, the discovery of glucose transporters in fibre cell membranes throughout the lens suggests that lens fibre cells can directly take up extracellular glucose in all regions of the lens (Merriman-Smith et al., 1999; Merriman-Smith et al., 2003; Lim et al., 2017). This regional distribution of glucose transporters is more consistent with the existence of an internal microcirculation system that has been proposed to use circulating fluxes of ions and water to drive the extracellular delivery of nutrients to the nucleus of the lens faster than can be achieved by passive diffusion alone (Donaldson et al., 2001; Mathias et al., 2007). The existence of this microcirculation system and its role in actively maintaining the lens’s optical properties has been established using a variety of techniques (Gao et al., 2011; Vaghefi et al., 2011; Candia et al., 2012; Vaghefi et al., 2015). Moreover, MRI studies that tracked the extracellular delivery of contrast agents to different lens regions show that solute delivery to the lens nucleus did indeed occur faster than would be expected by passive diffusion alone and that this delivery was abolished by inhibiting the microcirculation system (Vaghefi and Donaldson, 2018). Although these studies did not specifically study glucose delivery, they suggest that small solutes like glucose enter the lens at the anterior and posterior poles via an extracellular pathway that preferentially delivers them to the lens nucleus where they can then be taken up into fibre cells by the transporters known to be expressed in these cells.

Glucose uptake is mediated by two major protein families: the GLUT family that mediates the facilitative diffusion of glucose; and the SGLT family that utilises the energy stored in the Na^+^ electrochemical gradient to actively accumulate intracellular glucose (Wright et al., 2011). GLUT and SGLT isoforms have different affinities for glucose, allowing it to be taken up from extracellular environments with varying glucose levels. The presence and spatial distributions of GLUTs and SGLTs in the lens have been investigated at the mRNA and protein level in a number of species, including mouse, rat (Merriman-Smith et al., 1999; Merriman-Smith et al., 2003), bovine, and humans (Lim et al., 2017). Both GLUT1 and GLUT3 have been found in rodent and human lenses, while in the bovine lens, only GLUT1 has been found by Western blotting (Lim et al., 2017). It is clear that GLUTs are essential for lens transparency since the knockout of GLUT1 in a mouse model led to lens cataract formation (Swarup et al., 2018).

**FIGURE 1:**
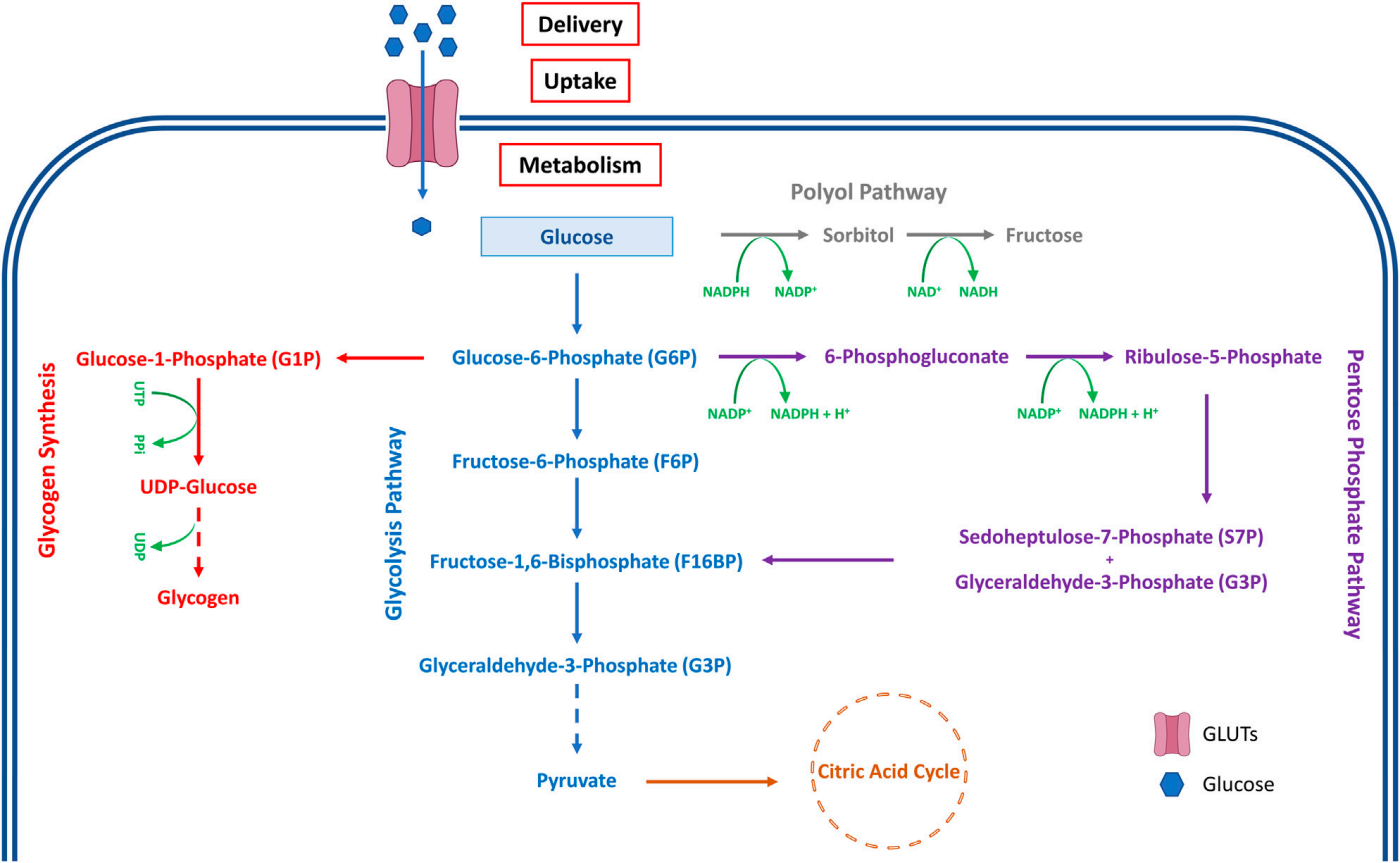
Major pathways of glucose metabolism in the lens.

Once inside the cell, glucose can be utilised in many metabolic processes to release energy in the form of ATP, which is required to maintain the structural integrity and, therefore, transparency of the lens (Vrensen, 1991; Donaldson et al., 2017). In the lens, it is primarily metabolised by three pathways (Figure 1), glycolysis (Kinoshita, 1955), the pentose phosphate pathway (hexose monophosphate shunt) (Giblin et al., 1981), and the polyol pathway (Dvornik et al., 1973; Kinoshita, 1990). Glycolysis is essential to maintain lens physiological function and tissue transparency over many decades of life since perturbations to major enzyme-mediated steps in glycolysis result in lens swelling and cataract (Kinoshita, 1955; Hejtmancik et al., 2015). The lens has a similar glycolytic pathway to other tissues, and within the lens, it was traditionally believed that the primary location of glucose metabolism in the lens was the epithelium (Swarup et al., 2018). For example, aerobic glycolysis and metabolism via the citric acid cycle are possible only in the lens epithelium and peripheral fibre cells that contain cell nuclei, mitochondria, and other cellular organelles. However, the aerobic metabolism performed in the lens only accounts for ∼20–30% of the total lens ATP production, while only consuming ∼3% of the glucose supplied to the lens (Hockwin et al., 1971; Trayhurn and Van Heyningen, 1972). The remaining 70% of lens glucose metabolism is carried out under anaerobic conditions (Bron et al., 1993), which produces lactate that is thought to contribute to a measurable pH gradient from the periphery to the centre of the lens (Bassnett and Duncan, 1986).

The environment in which the lens sits also plays a role in determining metabolic function and lens transparency. The lens sits in a mildly hypoxic environment in the normal eye, yet it can actively control its ion balance, maintain ATP levels, and synthesise proteins. However, exposure of cultured lenses to oxidative stress induced by exposure to hydrogen peroxide (Giblin et al., 1985) or hyperbaric oxygen (Giblin et al., 1988) stimulates the pentose phosphate pathway by increasing hexokinase activity (Hejtmancik et al., 2015). Despite not generating a large amount of ATP, the pentose phosphate pathway is essential because it uses the enzyme glucose-6-phosphate dehydrogenase to synthesize substantial amounts of NADPH (Figure 1) that is used by glutathione reductase to maintain redox balance in the lens by regenerating GSH from oxidised GSSG (Giblin et al., 1981). In addition, NAPDH is used by the third glucose metabolic pathway in the lens, the polyol (sorbitol) pathway. This route was initially described by van Heyningen in 1959, who observed the accumulation of polyols in the lens (Dvornik et al., 1973). In this pathway, sorbitol is formed by aldose reductase using NADPH as a cofactor, which is then converted to fructose by a second enzyme, polyol (sorbitol) dehydrogenase, using nicotinamide adenine dinucleotide (NAD^+^) as a coenzyme (Figure 1). Under normal physiological conditions, almost one-third of the glucose entering the human lens is metabolised through this sorbitol pathway (Jedziniak et al., 1981). In the human lens, the majority of aldose reductase, and its activity, is present predominantly in the epithelium (Jedziniak et al., 1981). Elevated levels of sugars such as glucose in the blood and AH, as occurs in diabetes (Davies et al., 1984), have been linked to hyperglycaemia-related changes to the lens and cortical cataract formation (Bron et al., 1993). Sorbitol is osmotically active, and therefore an overabundance of sorbitol can draw additional water inside the cell, causing cell swelling and damage, which disrupts the ordered lens cell architecture to cause light scattering. While this mechanism has been demonstrated in species such as dogs (Murata et al., 2001) and rodents (Kador et al., 2007), aldose reductase activity in the human lens is lower (Jedziniak et al., 1981), and its role in the aetiology of diabetic cataract is less clear.

Not only do there appear to be important differences in the activity of metabolic pathways between species, recent molecular screening also suggests that there are some important differences in the expression of GLUTs and SGLTs between rat, bovine and human lenses. Due to the unpredictable supply of human lenses that can be used in functional experiments, the bovine lens may be a good model to study lens function and cataract formation. Therefore, a previously established normoglycaemic model of *ex vivo* bovine lens incubations that utilises stable isotopically-labelled (SIL) glucose (Zahraei et al., 2020) has been characterised in the current study. Glucose utilisation and metabolite transport were traced through time by MALDI (Matrix-assisted laser desorption/ionisation) IMS (imaging mass spectrometry), with SIL metabolite validation performed via gas chromatography-mass spectrometry (GC-MS). In addition, proteomics was used to confirm the presence of GLUTs in the bovine lens and immunohistochemistry to localise GLUT transporters that mediate uptake of glucose from the humours.

MALDI-IMS showed the initial sites of SIL glucose uptake and metabolism occurred predominantly in the epithelial and DF cells located at the lens equator. However, longer incubations of up to 20 h showed that SIL metabolites were able to penetrate to other regions of the lens, including the lens nucleus. Interestingly, a proteomics approach detected both GLUT1 and GLUT3 in normal bovine lenses, and their relative abundance changed as epithelial cells differentiated into fibre cells. Taken together, this work establishes the metabolomic and proteomic profiles of glucose metabolism of the normal lens, which can be used as a baseline for future spatially resolved screening of the metabolic changes that occur in diabetic cataract.

## Materials and Methods

### Tissue and Reagents

Commercially available primary antibodies that were used for immunohistochemistry (IHC) to detect glucose transporters in the bovine lens are detailed in Supplementary Table S1. The secondary antibody goat anti-rabbit IgG-Alexa Fluor^®^ 488 and the nuclei cell marker DAPI were both purchased from ThermoFisher Scientific (Waltham, MA, United States). The cell membrane marker WGA-Alexa Fluor 594 was purchased from Life Technologies (Carlsbad, CA). Phosphate buffered saline (PBS, 10 mM phosphate buffer, 2.7 mM potassium chloride and 137 mM sodium chloride, pH 7.4) was obtained from Sigma-Aldrich (St Louis, MO). All solvents used were HPLC grade, and other chemicals were purchased from Sigma-Aldrich (St Louis, MO).

### *Ex vivo* Lens Incubations

Fresh bovine eyes were collected from a local abattoir (Auckland Meat Processors, Ltd.,). Lenses were dissected from ocular globes anteriorly and directly incubated in 12 ml of pre-warmed isotonic artificial aqueous humour (AAH, 120 mM NaCl, 0.5 mM MgSO_4_, 4.5 mM KCl, 26 mM NaHCO_3_, 2 mM CaCl_2_, 1 mM NaH_2_PO_4_.H_2_O, 10 mM HEPES, pH 7.2–7.4, 290 ± 5 mOsm/L) containing 5 mM stable isotopically-labelled (SIL) glucose [(U-^13^C_6_) glucose, Mw = 186.110], with un-labelled AAH containing glucose (^12^C_6_H_12_O_6_, Mw = 180.063) as a control. Lenses were incubated for times that ranged from 5 min to 20 h at 37°C in 5% CO_2_ using a tissue incubator (Heracell VIOS 160i CO_2_ incubator, ThermoFisher Scientific, Waltham, MA, United States). Following incubation, samples were rinsed three times in 20 ml isotonic AAH to remove any SIL glucose on the lens tissue surface and then stored at −80°C (Zahraei et al., 2020).

### Tissue Preparation for MALDI-IMS

Frozen lenses were mounted on cold chucks using Cryomatrix™ compound (ThermoFisher Scientific, Waltham, MA, United States). A cryostat (Leica CM3050S, Leica Microsystems GmbH, Wetzlar, Germany) was used to cut axial sections with a thickness of 20 μm. Sections were then immediately mounted on cooled double-sided carbon tape (ProSciTech, Kirwan, Australia) attached to non-conductive glass slides (PINK COLORFROST, LabServ, NZ). Collected sections were stored in a vacuum desiccator for at least 1 h and equilibrated to room temperature immediately before the application of the matrix. A N-(1-naphthyl) ethylenediamine dihydrochloride (NEDC) solution (7 mg/mL in 90% EtOH) containing an internal standard (IS) 3-OMG [(M + Cl)^−^ = *m/z* 229.0473] was applied using a TM-Sprayer (HTX Technologies, Carrboro, NC). Following matrix/IS spraying, slides were stored in a vacuum desiccator until used for data acquisition (Zahraei et al., 2020).

### MALDI Imaging Mass Spectrometry

MALDI-IMS of whole bovine lens sections was acquired using a raster step size of 150 μm on SolariX XR 7T FT-ICR instrument using FTMS Control v2.2.0 and flexImaging v5.0 software (Bruker Daltonics, Billerica, MA). Higher spatial sampling using a raster step size of 30 μm was used to assess SIL glucose uptake in specific lens cortical regions. Tissues were analysed in negative-ion mode using adjacent sections and data acquired in the mass-to-charge ratio (m/z) range of *m/z* 100–1,000 with a resolving power (m/Δm) of 66,000 at *m/z* 400. The laser scanned across sections in x and y, with 200 laser shots summed for each position at a repetition rate of 1 kHz. While laser power was optimised for each data set, the laser beam dimensions were matched to the raster step size by setting it to “medium” for all overview images collected and “small” for higher spatial sampling data. Before the acquisition, an external *m/z* calibration was applied using red phosphorus in MALDI MS negative ion mode. Since sections from all timepoints could not fit into the instrument simultaneously, samples were run immediately following each other to minimise sampling variability. Lens sections were also sampled in random order to avoid sampling order artefacts. Data sets from all incubation timepoints were then imported into SCiLS Lab 3D (version 2020 Pro, SCiLS GmbH, Bremen, Germany), and combined into a single data set. MALDI images were plotted at the theoretical *m/z* ± 0.005 Da with normalisation to the IS signal without de-noising function. Identities were assigned based on accurate mass matching, isotope distribution analysis, and comparison to GC-MS data where possible (see below) (Zahraei et al., 2020). A subset of metabolite MALDI images was plotted as 3D surface plots, and intensity profiles generated to show signal intensity changes across lens sections using ImageJ software (v1.49 m, National Institutes of Health, United States).

### Immunohistochemistry and Confocal Microscopy

Normal bovine lenses were fixed in 2% paraformaldehyde that contained 0.01% glutaraldehyde for 72 h immediately following removal from the ocular globe. Prior to labelling 16 µm thick axial lens sections with primary antibody, sections were permeabilised in 0.5% Tween 20^®^ for 20 min before incubation in blocking solution (3% BSA, 3% Normal Goat Serum, in ×1PBS) for 1 hour. Sections were then incubated overnight in GLUT1 or GLUT3 primary antibodies (Supplementary Table S1) diluted 1:200 in blocking solution. After incubation, sections were washed in ×1PBS and incubated in a secondary antibody conjugated to Alexa Fluor-488. Sections were then washed and incubated with a mixture of Alexa Fluor WGA-594 and DAPI diluted at 1:100 and 1:1,000, respectively, in PBS for 1 h at room temperature to visualise the lens cell morphology (Lim et al., 2017). Following a series of washes in PBS, sections were mounted in Vectashield (Vector Laboratories, CA, United States ) and imaged using a × 60 objective lens on an Olympus FV1000 confocal laser scanning microscope (Olympus Corporation, Tokyo, Japan) with FluoView 2.0c software. Digital images (2048 × 2048 pixels) were collected using optimised gain settings that captured the entire dynamic range of the signal at each tissue location. Images were pseudo-coloured using Adobe Photoshop software (v23.1.1, Adobe Inc., San Jose, California).

### Tissue Microdissection for Metabolite and Protein Analysis

Bovine lenses (either fresh, untreated or cultured with SIL glucose as above, *n* = 6) were micro-dissected with tweezers. The capsule containing the lens epithelium was peeled away from the underlying lens fibre cells. An 8 mm surgical punch was used to collect samples from central, peripheral and equatorial regions of the epithelium, and samples pooled for protein preparation. Samples from peripheral fibres were also collected by using tweezers to peel off cellular material from the periphery of the decapsulated lenses. The denser underlying inner cortical tissue was removed to expose the hard, central nucleus, and tweezers were used to collect fibre cells from this central lens region.

### Protein Preparation and SDS-PAGE

Tissues from each micro-dissected lens region were homogenised in an ice-cold lysis buffer (5 mM Tris, pH 8.0, 5 mM EDTA, 5 mM EGTA) that contained a protease inhibitor cocktail (Roche, Mannheim, Germany), using a hand-held homogeniser (Ultra-Turrax^®^, IKA-Werke, Staufen, Germany). Following centrifugation at 15,000 g for 30 min at 4°C, the pellet was separated from the supernatant. Both the pellet and supernatant were loaded and separated on an 10% SDS PAGE gel (Mini-PROTEAN^®^ Bio-Rad Laboratories Inc., Hercules, California, United States), following standard protocols.

### Gel-Based Proteomics (GeLC/MS)

A prominent band at the Mw 53 kDa, corresponding to the molecular weight of GLUTs, was cut from samples of each collected region using a 6 × 2 mm punch. The gel bands were placed in different 1.5 mL Eppendorf tubes and diced into cubes. The gel bands were prepared for LC/MS analysis following standard protocols. Briefly, the bands were washed and de-stained with 50% acetonitrile in ammonium bicarbonate buffer (50 mM). Following de-staining, the bands were dehydrated with acetonitrile, reduced using dithiothreitol and concomitantly alkylated with iodoacetamide. Subsequently, the bands were dehydrated again and were finally subjected to enzymatic digestion using 12.5 ng/μl porcine trypsin (Promega Corp., Madison, Wisconsin) in a temperature-controlled microwave at 45°C for 1 h. The digest was acidified to halt digestion, and peptides were injected onto a 0.3 × 10 mm trap column packed with Reprosil C18 media (Dr. Maisch HPLC GmbH, Ammerbuch, Germany) and desalted for 5 min at 10 μl/min before being separated on a 0.075 × 200 mm picofrit column (New Objective, Inc., Littleton, Massachusetts, United States) packed in-house with 3 μm Reprosil C18 media. A 30%–50% solvent B gradient was applied at 300 nl/min using a NanoLC 400 UPLC system (Eksigent Technologies, Redwood City, California), where solvent A was 0.1% formic acid in water, and solvent B was 0.1% formic acid in acetonitrile. The picofrit spray was directed into a TripleTOF 6,600 Quadrupole-Time-of-Flight mass spectrometer (AB Sciex LLC, Framingham, Massachusetts) scanning from 300 to 2000 m*/z* for 200 ms, followed by 40 ms MS/MS scans on the 35 most abundant multiply-charged peptides (*m/z* 80–1,600). The mass spectrometer and HPLC system were controlled by the Analyst TF 1.7 software package (AB Sciex LLC, Framingham, Massachusetts, United States). The resulting MS/MS data were searched against a database comprising Uniprot bovine entries appended with a set of common contaminant sequences using ProteinPilot version 5.0 (AB Sciex LLC, Framingham, Massachusetts, United States). Search parameters were as follows: Sample Type = Identification; Search Effort = Thorough; Cys Alkylation = Iodoacetamide; Digestion = Trypsin. The peptide summary exported from ProteinPilot was further processed in Excel (v2102, Microsoft Corp., Albuquerque, New Mexico) using a custom macro to remove proteins with Unused Scores below 1.3, to eliminate inferior or redundant peptide spectral matches, and to sum the intensities for all unique peptides from each protein.

### GC-MS Analysis

Micro-dissected fibre cell regions were placed in separate falcon tubes containing 2 ml of 90% MeOH. Samples were agitated and homogenised in 90% MeOH for 45 min at 4°C. The remaining cell debris was removed via centrifugation at 16,000 g for 20 min at 4°C in an Eppendorf 5402 centrifuge, and supernatants containing extracted small molecules were kept at −80°C until required for analysis. Samples were then dried in a Speed-vac Concentrator (Thermo SPD121P, ThermoFisher Scientific, Waltham, MA, United States) and underwent automated trimethylsilyl derivatisation in preparation for analysis to identify SIL sugars using an Agilent 7890B gas chromatograph coupled to a 5977A inert mass spectrometer (Zarate et al., 2017). Instrument parameters for GS-MS analysis were based on Villas-Bôas et al. (2006) and analysis was performed according to Zahraei et al. (2020). GC-MS data were converted into.cdf files by the function provided in the Automated Mass Spectral Deconvolution and Identification System (AMIDS). A sub-library of the spectral database was generated by searching the QC samples against the NIST17 database. The identification result was then combined with an amino acid and sugar standard reference spectral library. Spectra of SIL molecules of interest were annotated and curated manually with the assistance of the NIST17 database.

## Results

We have used a multi-omics approach to characterise the delivery, uptake, and metabolism of glucose in different regions of the bovine lens. Spatial resolution at multiple scales has been preserved using tissue punches and confocal imaging for protein analysis and microdissection and MALDI-IMS for metabolite analysis.

### Glucose Delivery

**FIGURE 2:**
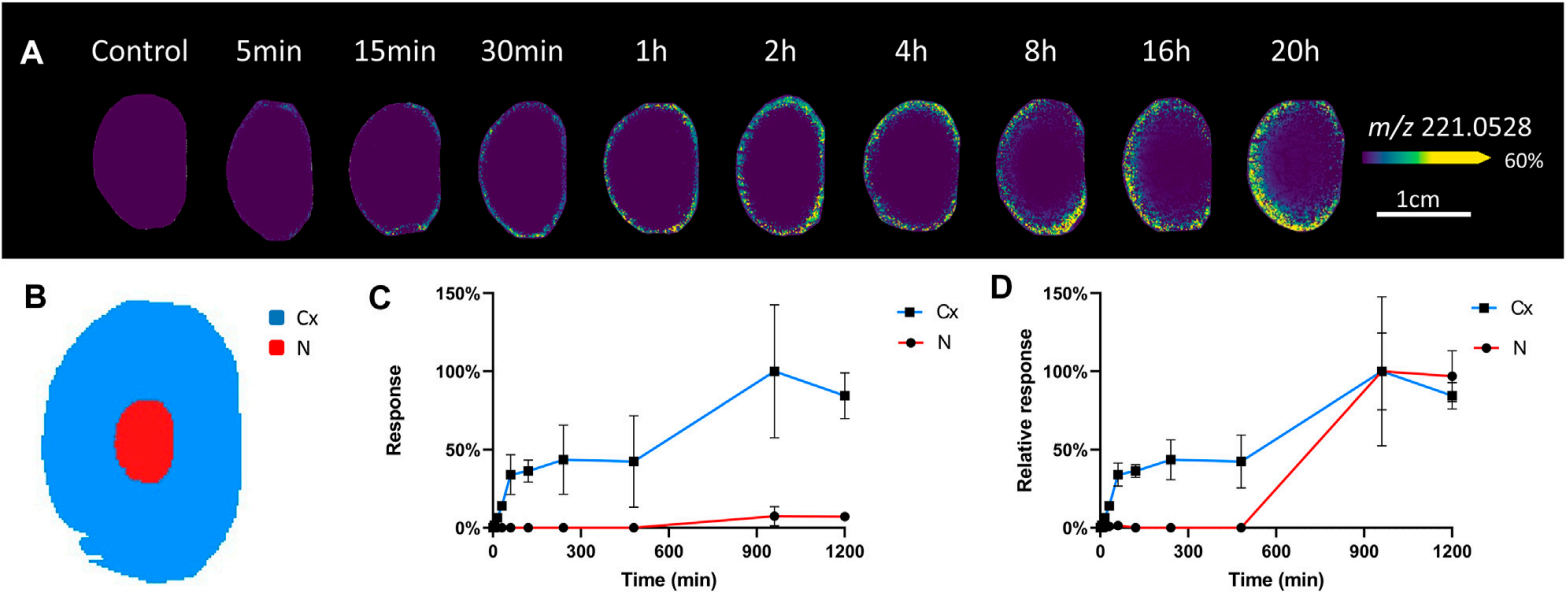
Visualisation of SIL glucose uptake in incubated bovine lenses. **(A)** A series of MALDI images of axial sections taken from bovine lenses organ cultured in AAH containing 5 mM SIL glucose for 5 min to 20 h showing regional differences in SIL glucose (*m/z* 221.0528) uptake. Control lenses incubated in normoglycaemic AAH containing non-labelled glucose showed no signal at *m/z* 221.0528. Lenses orientated with anterior surface to the right. **(B)** Schematic diagram indicating the two regions [cortex (Cx) = blue, nucleus (N) = red] from which SIL glucose signals were extracted for relative quantification and comparison. Relative intensity plots of *m/z* 221.0528 from each lens region showing signal increase over time normalised to maximal signal in the dataset. **(C)**, and to maximal signal within each lens region **(D)**.

To initially assess the pattern of glucose uptake, *ex vivo* bovine lenses were incubated in AAH containing a normoglycaemic level of SIL glucose [(U-^13^C_6_) glucose], which due to its shift in mass (+6 amu) can be detected as a *m/z* signal in the mass spectrum that is distinct from the spectrum of endogenous glucose (^12^C_6_H_12_O_6_) and other isobaric metabolites. Initially, incubations from 5 min to 20 h were used to assess global uptake (Figure 2). A signal detected at *m/z* 221.0528 in the MALDI-IMS data was assigned as SIL glucose [(M + Cl)^−^] since it was within 2 ppm of the predicted *m/z* of SIL glucose (Table 1), was not present in control lenses incubated in AAH containing unlabelled glucose (Figure 2A), and was previously not detected in control samples analysed by GC-MS (Zahraei et al., 2020). Closer inspection of the spatial distribution of timepoints up to 1 h incubation showed initial uptake of SIL glucose occurred at the equator and then spread around the entire periphery of the lens. This signal was more uniformly distributed in the lens outer cortex at 2–8 h, while at 16 and 20 h SIL glucose signal had reduced markedly in the lens anterior region, and a very low signal was also detected in the lens nucleus. Digital dissection of three replicates of each timepoint into cortex and nucleus (Figure 2B) showed signal intensity changes for *m/z* 221.0528 were most prominent in the cortex, but the signal was also detectable in the nucleus at longer time points even though it was of low intensity relative to the cortex (Figure 2C). When intensity plots were normalised to the maximum signal in each region, the SIL glucose signal was present in the nucleus after 16 h of incubation in SIL glucose (Figure 2D).

**TABLE 1:** Predicted and observed SIL metabolite m/z.

SIL metabolite identity	Adduct	Predicted m/z	Observed m/z	Error (ppm)
Glucose	[M + Cl]^−^	221.0523	221.0528	1.8
Glucose-6-phosphate	[M–H]^−^	265.0425	265.0432	2.6
Fructose-1,6-bisphosphate	[M–H]^−^	345.0089	345.0106	4.9
Sedoheptulose-7-phosphate	[M–H]^−^	295.0531	295.0510	7.1
UDP-Glucose	[M–H]^−^	571.0678	571.0722	7.7
Sorbitol	[M + Cl]^−^	223.0680	223.0686	2.6

**FIGURE 3:**
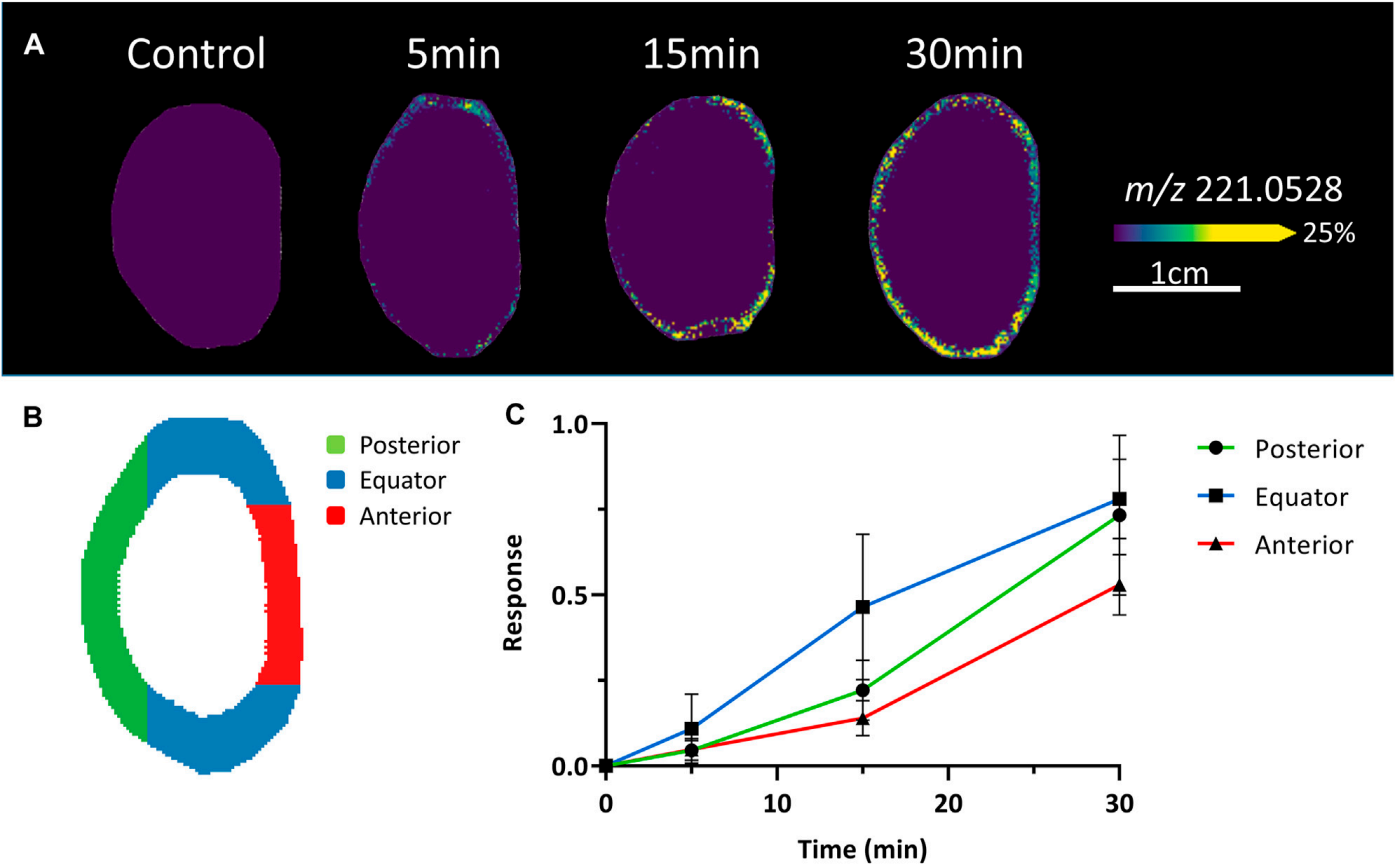
Comparison of initial glucose uptake at the surface of the lens. **(A)** A series of MALDI images of axial sections taken from bovine lenses organ cultured in AAH containing 5 mM SIL glucose for 5–30 min showing initial differences in SIL glucose (*m/z* 221.0528) uptake at the surface of the lens. Control lenses incubated in normoglycaemic AAH containing non-labelled glucose showed no signal at *m/z* 221.0528. Lenses orientated with anterior surface to the right. **(B)** Schematic diagram indicating the different regions (anterior = red, equator = blue, posterior = green) of the lens surface from which SIL glucose signals were extracted for relative quantification and comparison. **(C)** Intensity plots from the different regions shown in *B* indicate that the initial uptake of SIL glucose at the surface of the lens occurs predominately in the equatorial region, which contains both equatorial epithelial cells and elongating fibre cells, relative to the anterior and posterior regions of the lens surface.

Because of the low signal intensity of the SIL glucose signal in the deeper lens regions, we chose to focus on the initial (5–30 min) uptake of glucose in the epithelium and peripheral fibre cells (Figure 3). After a 5 min incubation, the SIL glucose signal (*m/z* 221.0528) was most intense around the equatorial region, with a higher signal in the epithelial and outer cortical region just anterior to the equator. Signal intensified after 15 min, extending both posteriorly and anteriorly, and continued to spread to the entire lens periphery after 30 min (Figure 3A). This preferential uptake of SIL glucose at different lens surface locations was quantified in the equatorial, anterior, and posterior regions (Figure 3B) and average changes in intensity were plotted as a function of time for three lenses (Figure 3C). This analysis showed SIL glucose uptake from the surrounding media was strongest in the equatorial region of the lens, before being detected in the anterior and posterior regions of the lens (Figure 3C). Together these results suggested a differential affinity or rate of glucose uptake occurred in the equatorial region of the lens that consisted of equatorial epithelial cells and DF cells.

### Glucose Uptake

**FIGURE 4:**
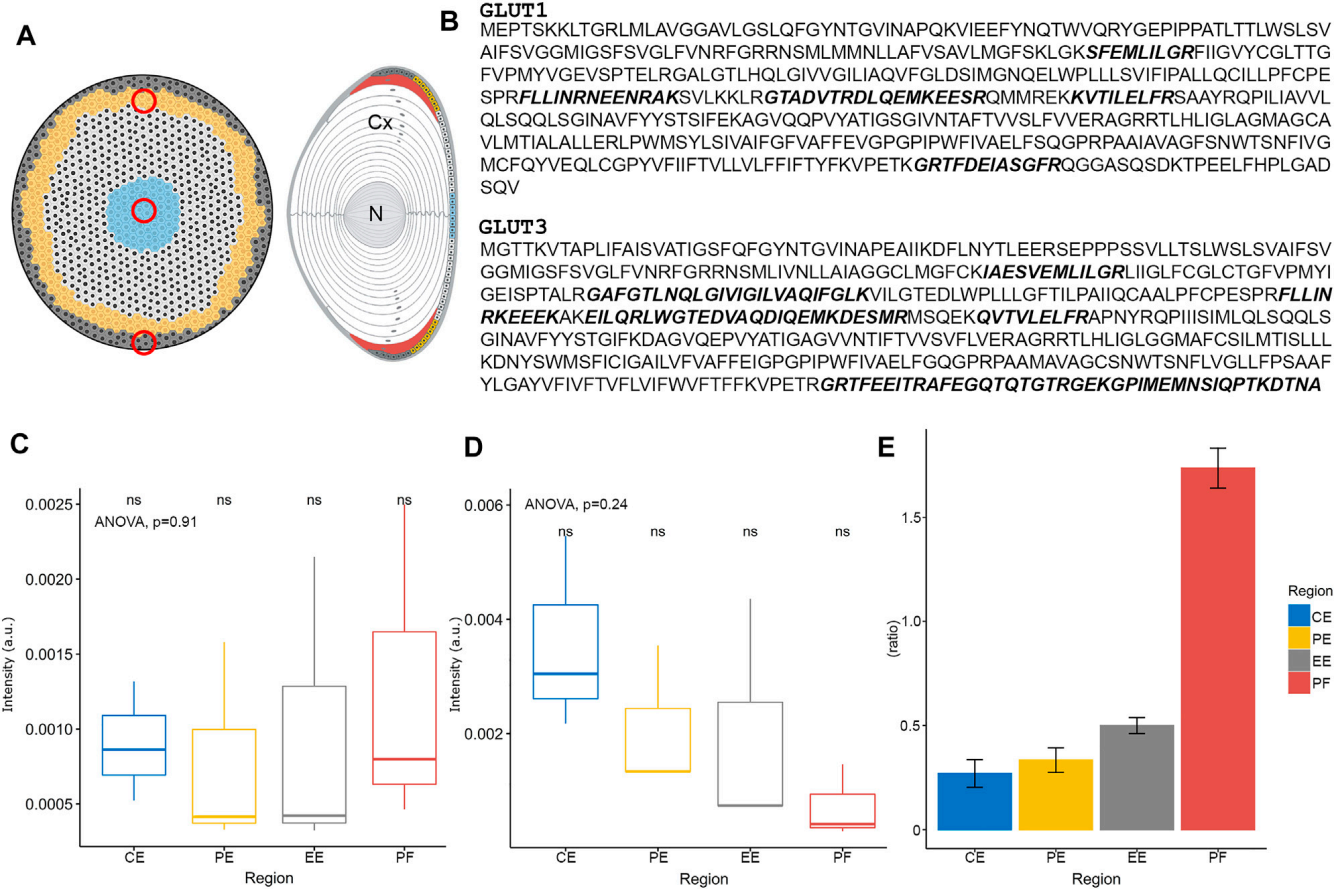
Spatial proteomics of GLUTs in the lens epithelium and outer cortex. **(A)** Schematic diagram of bovine lens epithelial cell flat mount (left) and axial section (right) indicating regions (red circles) where tissue was collected for subsequent proteomic analysis. Cx = cortex, N = nucleus. **(B)** Amino acid sequences of bovine GLUT1 and GLUT3. Peptides detected by proteomic analysis are indicated in bold, italic, and represent a combination of some 11 and 16 peptide fragments collected from all tissue regions for GLUT1 and GLUT3, respectively. **(C)** Relative quantitation of GLUT1 protein levels in the different lens epithelium and outer fibre cell regions, normalised to vimentin. **(D)** Relative quantitation of GLUT3 protein levels in the different lens epithelium and outer fibre cell regions, normalised to vimentin. **(E)** GLUT1:GLUT3 ratio in different bovine lens tissue regions. CE = central epithelium (*blue*), PE = peripheral epithelium (*yellow*), EE = equatorial epithelium (*grey*), PF = peripheral fibre cells (*red*). ns = not statistically significant as tested by ANOVA.

To determine whether the observed pattern of SIL glucose uptake was due to the presence of GLUTs at different quantities in different lens regions, proteomics with relative quantitation was performed on membrane protein fractions prepared from the different tissue regions (Figure 4). In the first instance, tissue from epithelial explants (Figure 4A, *left*) and the cortical and nuclear regions (Figure 4A, *right*) of the lens were sampled to determine the presence or absence of different GLUT isoforms in a non-quantitative manner. Cell membrane proteins from each preparation were separated via SDS-PAGE, and the gel band corresponding to the molecular weight of GLUTs (∼53 kDa) was cut, trypsinised, and peptides separated via reverse-phase HPLC and detected via MS/MS. Interestingly, this approach detected peptides from both GLUT isoforms 1 and 3 in each lens region (see Supplementary Figure S1). Further targeted mass spectrometry could be used in the future to determine relative amounts in the different lens fibre cell regions. Next, to focus on further understanding, the pattern of initial SIL glucose uptake revealed by MALDI-IMS in peripheral lens tissues, tissue punches from central, peripheral, and equatorial epithelium regions were collected and compared to outer cortical fibre cells harvested from the lens equatorial region (Figure 4A), using the above Gel-LC approach.

GLUT1 was detected in all lens epithelial regions, and in lens fibre cells, with a combined peptide number of 11 across all regions (Figure 4B, *upper*), confirming the previous detection in the bovine lens by Western blot analysis (Lim et al., 2017). Several other proteins were detected in this band, with almost complete coverage of vimentin (*data not shown*), an intermediate filament protein that forms part of the cytoskeleton which is known to be expressed in the lens epithelium and DF cells (Sandilands et al., 1995). Consistent with the less spatially resolved sampling above, GLUT3 was also detected in all lens epithelial regions and outer lens fibre cells, with 16 peptides detected across all analysed regions (Figure 4B, *lower*). When GLUT signals were normalised to the vimentin signal to control for different cell densities that are present in different lens epithelium regions (Wu et al., 2015), the levels of GLUT1 remained relatively constant (Figure 4C). In contrast, the levels of GLUT3 trended down from central to the equatorial epithelium (Figure 4D); however, these differences were deemed not statistically significant by ANOVA. Relative quantitation of GLUT1:GLUT3 showed that GLUT1 was less abundant than GLUT3 in all regions of the epithelium. However, in outer cortical fibre cells, that ratio reversed and GLUT1 was more abundant (Figure 4E). To further confirm the presence of GLUTs in the bovine lens, and determine their subcellular location, immunofluorescence confocal microscopy was utilised.

**FIGURE 5:**
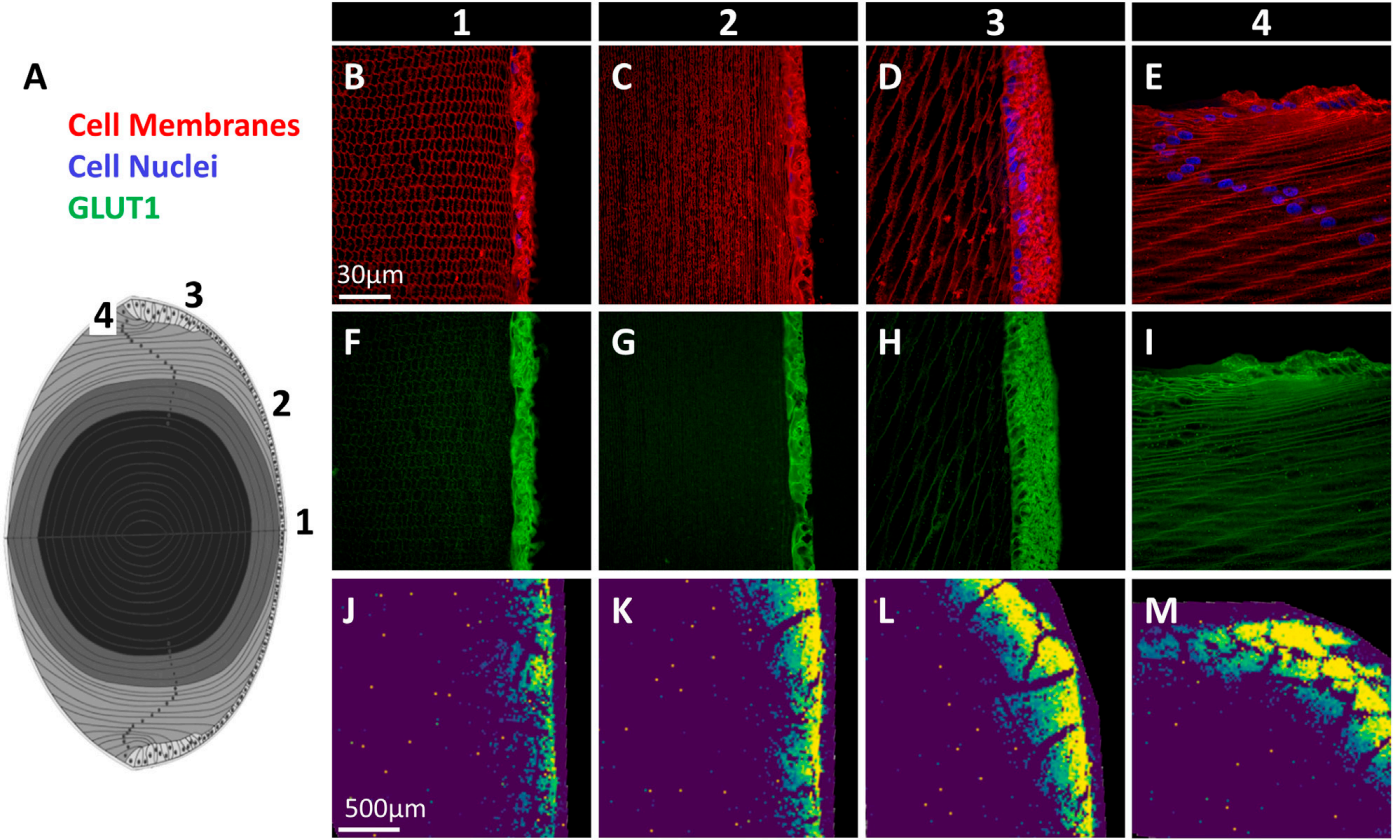
Immunofluorescence mapping of GLUT1 in bovine lens. **(A)** Schematic diagram of lens axial section with locations of imaged regions indicated (1–4). **(B–E)** Cell membrane marker (*red*) and cell nuclei (*blue*) signal from marked regions in panel **(A)** show epithelial and fibre cell morphology. **(F–I)** GLUT1 immunolabelling from corresponding regions. **(J–M)** MALDI images of SIL glucose (*m/z* 221.0528) uptake in bovine lenses indicating the extent of SIL glucose uptake mediated by GLUTs in each region.

Axial lens sections were triple labelled with the cell membrane marker WGA (*red*), DAPI to mark cell nuclei (*blue*), and antibodies against GLUT1 or GLUT3 (*green*), and representative images of the epithelium and underlying fibre cells collected from designated regions from the anterior pole to equator (Figure 5A). For GLUT3, using an antibody targeting the C-terminal tail of the protein, no labelling was observed (*data not shown*). This was surprising since peptides from the C-terminus of GLUT3, the epitope for the majority of trialled GLUT3 antibodies (see Supplementary Table S1), were detected by mass spectrometry (see Figure 4B). However, this is consistent with previous Western blot analysis that was unable to detect GLUT3 in bovine lenses (Lim et al., 2017). In contrast, GLUT1 was detected predominantly in the cell membrane of epithelial cells at the anterior pole, with a low level of labelling in underlying lens fibres (Figures 5B,F). A similar pattern was detected in lens epithelial cells positioned mid-way between the anterior pole and equator (Figures 5C,G). In epithelial cells located in the peripheral epithelial zone, GLUT1 immunolabelling was more diffuse, and some punctate labelling was also detected in the underlying lens fibres (Figures 5D,H). Finally, at the lens equator where lens epithelial cells are differentiating and elongating (i.e., the transitional zone), pronounced cell membrane labelling for GLUT1 was detected, in addition to punctate cytoplasmic labelling (Figures 5E,I). A low level of signal equivalent to anterior pole labelling levels was also detected in lens fibre cells at the posterior pole (*data not shown*). MALDI-IMS of sections from bovine lenses incubated for 30 min in AAH containing SIL glucose showed uptake of *m/z* 221.0528, assigned SIL glucose, in regions where GLUT1 immunolabelling was detected (Figures 5J–M). The spatial correlation of GLUT1 labelling and SIL glucose signal suggests that GLUT1 mediates the uptake of glucose in these specific regions of the normal bovine lens cortex. However, a role for GLUT3 cannot be ruled out, and genetic tools and/or pharmacological interventions could be used in the future to determine the relative roles of GLUTs 1 and 3 in glucose uptake in the bovine lens cortex.

### Glucose Metabolism

**FIGURE 6:**
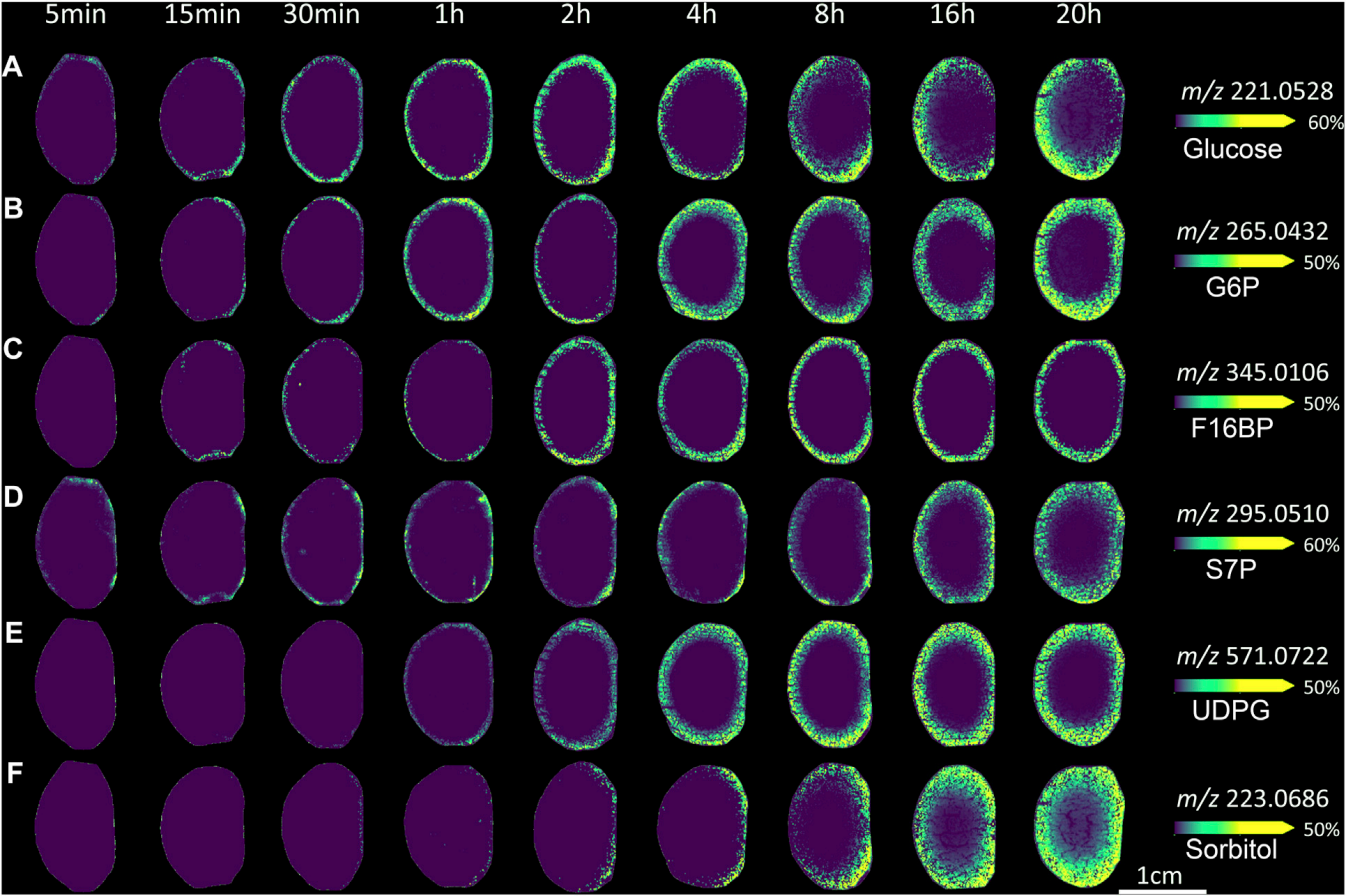
Visualising lens glucose metabolism and transport in *ex vivo* bovine lens. MALDI images from axial sections taken from bovine lenses organ cultured for varying times in AAH containing 5 mM SIL glucose. Lenses orientated with anterior surface to the right. **(A)** *m/z* 221.0528 assigned to SIL glucose. **(B)** *m/z* 265.0432 assigned SIL to glucose-6-phosphate, as a marker of glycolysis. **(C)** *m/z* 345.0106 assigned to SIL fructose-1,6-bisphosphate, as a marker of glycolysis. **(D)** *m/z* 295.0510 assigned to SIL sedoheptulose-7-phosphate, as a marker of the pentose phosphate pathway. **(E)** *m/z* 571.0722 assigned to SIL UDP-glucose, as a precursor of glycogen synthesis **(F)** *m/z* 223.0686 assigned to SIL sorbitol, as a marker of the polyol pathway. G6P = glucose-6-phosphate, F16BP = fructose-1,6-bisphosphate, S7P = sedoheptulose-7-phosphate, UDPG = UDP-glucose.

Once taken up into the cell, glucose is rapidly metabolised to produce ATP to drive other signalling and metabolic processes and reducing equivalents such as NADH and NADPH. Since lens glucose is predominantly metabolised by glycolysis (to produce ATP), the pentose phosphate shunt (to produce NADPH), and the sorbitol pathway, analysis of the fate of SIL glucose was performed by mapping the distribution of SIL glucose metabolites in lenses incubated for up to 20 h in SIL glucose AAH (Figure 6). For reference, the pattern and time course of SIL glucose throughout the lens is shown again in Figure 6A. In addition to this SIL glucose signal, signals for SIL glucose metabolites from each of the three metabolic pathways for glucose were detected by MALDI-IMS (Figures 6B–F). The identities of these signals were validated through a combination of accurate mass matching, isotopic distribution comparison (Supplementary Figure S2) and previous GC-MS analysis of identically incubated lenses (Supplementary Figures S3–S5). The results for each pathway are summarised in Table 1 and presented below.

*The glycolytic pathway:* The initial step in glycolysis, and a number of other metabolic pathways, is the phosphorylation of glucose. After 5 min a very low signal was observed at *m/z* 265.0432, representing a SIL hexose-6-phosphate, which is likely glucose-6-phosphate, glucose-1-phosphate, fructose-1-phosphate, or potentially a mixture of these metabolites (Figure 6B). Previous GC-MS analysis supported the assignment of glucose-6-phosphate (G6P) to this signal (Zahraei et al., 2020). The signal at 5 min was localised to the same region of lens epithelium that takes up SIL glucose. In longer incubations, the signal for G6P distributed to the entire epithelium (by 15 min) and then throughout the entire peripheral cortex (at timepoints longer than 2 h). Finally, signal intensity was observed in the entire cortex after several hours of incubation. At 20 h, a low signal level was also detected in the nucleus.

Later steps in the glycolysis pathway result in the formation of fructose-1,6-bisphosphate (F16BP). A signal that matched the predicted *m/z* for SIL F16BP was detected at *m/z* 345.0106 (Figure 6C). It was detected at very low levels after 5 min of incubation and was restricted to the outer cortical fibre cells even after several hours of incubation. These relatively low signal levels may indicate either a low concentration of F16BP, its relatively transient nature in the cell before being metabolised further or that the MALDI conditions used to detect it were not optimal. Previous reports have shown that this metabolite can also be detected by the MALDI matrices 9-AA (Dekker et al., 2015; Nye-Wood et al., 2016) and 1,5-DAN (Liu et al., 2014; Calvano et al., 2018). Therefore in the future, more detailed mapping of glycolysis pathways in the lens may require the parallel use of multiple matrices optimised for specific metabolites.

*The pentose phosphate pathway:* In the oxidative phase of the pentose phosphate pathway, glucose-6-phosphate is metabolised to ribulose-5-phosphate, and in the process, a number of reducing equivalents (NADPH) are produced, which help protect against oxidative stress by reducing oxidised glutathione. In the non-oxidative phase, ribulose-5-phosphate is metabolised in one of two ways to sedoheptulose-7-phosphate (S7P) (Lal et al., 1995; Szwergold et al., 1995). S7P is then cycled back via fructose-6-phosphate to undergo glycolysis, or can be further metabolised to erythrose 4-phosphate to be used in the synthesis of aromatic amino acids. A signal at *m/z* 295.0510, matching the predicted *m/z* for SIL S7P, was detected in all lenses incubated in SIL glucose (Figure 6D). This signal was generally limited to the most peripheral tissue regions, suggesting localisation to peripheral fibre cells and epithelium, with some stronger regions of signal generally on the lens anterior surface. At longer timepoints, a low signal was detected in the lens cortex, in a region where signals for both SIL glucose and SIL G6P were also detected (see Figures 6A,B).

A signal at *m/z* 571.0772 was also detected, although only at timepoints longer than 30 min (Figure 6E). This signal matched the predicted *m/z* for SIL UDP-glucose, a metabolite that is a precursor to glycogen and is also involved in the synthesis of glycosphingolipids. Signal for SIL UDP-glucose was relatively uniform around the entire lens, initially appearing in the peripheral cortex (at 1 h) before increasing in intensity and spreading throughout the entire lens cortex. However, no SIL glycosphingolipids were detected, potentially due to their concentration being below the limit of detection, that their biosynthesis takes longer than the 20 h incubation period used in this study, or that MALDI sampling conditions were not optimised for glycosphingolipid detection.

*The polyol pathway:* Another major metabolic pathway present in the lens is the polyol (sorbitol) pathway, which converts glucose to sorbitol through the enzyme aldose reductase. Aldose reductase is known to be present in the bovine lens (Del Corso et al., 1989), and its activity is elevated in hyperglycaemia (Srivastava et al., 2005). Under normal glucose conditions, a signal at *m/z* 223.0686 that matched the predicted *m/z* for SIL sorbitol was localised to the anterior lens surface after 30 min of incubation, indicating the formation of SIL sorbitol (Figure 6F). Over time, signal intensity for SIL sorbitol increased and was localised to the cortex at 8 h and longer timepoints. In addition, this signal was also detected in the lens nucleus at 16–20 h.

**FIGURE 7:**
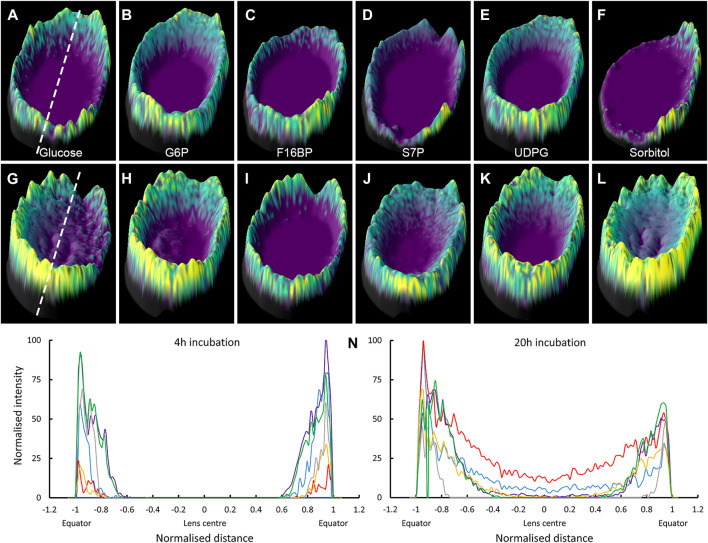
Signal intensity plots of SIL glucose and metabolites showing presence in nucleus over time. 3D intensity plots of SIL metabolite signals obtained in bovine lenses organ cultured for 4 h **(A–F)** and 20 h **(G–L)** in SIL glucose AAH. Lenses orientated with anterior surface to the right. **(A,G)** SIL glucose; *m/z* 221.0528, **(B,H)** SIL G6P; *m/z* 265.0432. **(C,I)** SIL F16BP; *m/z* 345.0106, **(D,J)** SIL S7P; *m/z* 295.0510, **(E,K)** SIL UDPG; *m/z* 571.0722, and (F, L) SIL sorbitol; *m/z* 223.0686. Dashed lines indicate regions used to generate signal intensity plots used to compare spatial differences in SIL metabolite accumulation after 4 h **(M)** and 20 h **(N)** of incubation in SIL glucose. *Blue* = SIL glucose, *red* = SIL sorbitol, *purple* = SIL G6P, *yellow* = SIL S7P, *grey* = SIL F16BP, *green* = SIL UDPG, G6P = glucose-6-phosphate, F16BP = fructose-1,6-bisphosphate, S7P = sedoheptulose-7-phosphate, UDPG = UDP-glucose.

To more clearly display the SIL metabolite signals detected in the lens nucleus, a subset of the two-dimensional MALDI images presented in Figure 6 were re-plotted as three-dimensional surface plots, where both the colour and height of the signal represents the signal intensity. MALDI images of the six SIL metabolites detected in both 4 and 20 h incubations were plotted, and intensity plots of each signal through the lens equator were generated (Figure 7). While it is clear the MALDI-IMS did not detect SIL metabolite signals in the lens nucleus after only 4 h of incubation, signals for SIL glucose, and SIL sorbitol in particular, were evident in the nucleus after 20 h of incubation. These results tend to suggest that SIL glucose is being delivered to the nucleus, but the levels detected are reduced due to the local metabolism of glucose to sorbitol which appears to be accumulated in the lens.

## Discussion

In this study, we have used MALDI-IMS to visualise regional differences in the delivery, uptake and metabolism of SIL glucose in the normal bovine lens and have used proteomic analysis (LC-MS/MS) and immunohistochemistry to validate our findings. Using this multi-pronged approach, we have shown that SIL glucose initially preferentially enters the lens at the equator (Figure 2) in a region where the abundance of GLUT1 relative to GLUT 3 increases (Figure 4). Furthermore, the SIL glucose taken up in these peripheral regions of the lens was available for use in the three main glucose metabolism pathways as indicated by the presence of metabolites of SIL glucose metabolism (Figures 6, 7). At longer time points, SIL glucose and a subset of SIL glucose metabolites were detected in the deeper regions of the lens, albeit at a lower level than what was observed in the outer cortex of the lens (Figures 6, 7), a result that is consistent with the identification of GLUT1 transporters in the nucleus of the lens (Supplementary Figure S1; Lim et al., 2017). These results show that our novel approach can be used to map the delivery, uptake, and metabolism of glucose in the bovine lens and that there are differences in how glucose is metabolised in the different regions of the lens.

The results obtained in this study using MALDI-IMS are consistent with those obtained using Raman spectroscopy (Hu et al., 2015), and NMR approaches (Cheng et al., 1991; Nakamura et al., 2003; Sawada et al., 2003). In these previous studies, glucose uptake and its metabolism to sorbitol were mapped in bovine and rabbit lenses organ cultured under hyperglycaemic conditions. In bovine lenses exposed to 50 mM for up to 4 days, glucose and sorbitol were restricted to the outer cortex, with glucose levels decreasing over time following initial uptake and a corresponding increase in cortical sorbitol levels (Sawada et al., 2003). In rabbit lenses incubated in 35.5 mM glucose for 28 h, glucose was found throughout the lens but was more abundant in the outer cortex, while sorbitol was abundant in the lens nucleus, and lactate was also detected (Cheng et al., 1991). While valuable, these studies suffered from poor spatial resolution, poor time resolution, and a limited number of metabolites were detected. Using our higher resolution MALDI-IMS approach, we have also shown that glucose and sorbitol were also present predominantly in the lens outer cortex, with the accumulation of sorbitol following the depletion of glucose signal as it was metabolised by the polyol pathway, albeit in a normoglycemic model.

Since signals for SIL glucose are not normally detected in the lens (Figure 2A), we have been able to use MALDI-IMS to visualise the delivery of SIL glucose to different regions of the lens. However, although this initial delivery of SIL glucose from the bathing solution to the lens must occur *via* the extracellular space, MALDI-IMS does not have the resolution to determine whether the SIL glucose signal subsequently detected in a specific region of the lens originates in either the extracellular and/or intracellular compartments. Because of the relative volumes of the two compartments, we believe that the majority of the SIL glucose signal we detect in any given region is predominately intracellular SIL glucose. Supporting this is the rapid appearance of a signal for SIL sorbitol, which is generated from SIL glucose by the cytoplasmic enzyme aldose reductase. Thus, while our MALDI-IMS approach can detect where in the lens SIL glucose accumulates, it cannot resolve how that SIL glucose was delivered to a specific region of the lens. To resolve extracellular delivery of SIL glucose, new generation MALDI instruments or alternative higher spatial resolution IMS approaches such as secondary ion mass spectrometry will be required (Niehaus et al., 2019; Spivey et al., 2019).

Despite the above caveats about the ability of MALDI-IMS to resolve extracellular delivery of SIL glucose to the lens we believe the early time points of SIL glucose in the peripheral epithelium and equatorial region (see Figure 3) can be attributed to direct uptake of SIL glucose from the extracellular space in what appears to be a localised region of enhanced uptake. A similar equatorial region of enhanced uptake of radioactive cysteine was found in monkey lenses (Sweeney et al., 2003). The enhanced SIL glucose signal in this region does not appear to be due to a higher density of cells, which is a known feature of the peripheral epithelial region (Wu et al., 2015), since the proteomic data was normalised to vimentin signal to account for this. These results suggest that in this region of the lens, where epithelial cells differentiate into fibre cells that undergo extensive elongation, increased levels of glucose are required to meet the high energy demands of DNA replication and protein and lipid synthesis. The observed initial preferential uptake of SIL glucose in this equatorial region can be explained by enhanced penetration of SIL glucose into the lens due to the relative lack of tight junctions between epithelial cells in this region which has been observed for the mouse lens (Goodenough et al., 1980). Alternatively, the observed uptake distribution could be due to an increasing cell membrane area in elongating cells, which has been proposed to explain the enhanced Na^+^/K^+^ ATPase channel current observed at the lens equator relative to the central epithelium (Gao et al., 2000).

Interestingly in the present study, both GLUT1 and GLUT3 were detected by our proteomic approach, a result that was consistent with a previous study that analysed samples collected from the bovine lens using laser micro-dissection (Wang et al., 2008). In contrast, antibody-based approaches to detect GLUT3 in the current study by immunofluorescence and previously by Western blot (Lim et al., 2017) failed to detect the protein in the bovine lens. While the precise epitopes of the GLUT3 antibodies used in this study are not known, a large portion of the C-terminal tail of GLUT3, which is the region targeted by most antibodies and two of the antibodies trialled here (see Supplementary Table S1), was detected by LC-MS/MS (see Figure 4B). Possible reasons that an antibody will not bind to a target sequence include inaccessibility, either through protein structure or interaction with other proteins, post-translational modification (PTM) to amino acid(s) in the epitope, such as phosphorylation, or low homology between the sequence used to raise the antibody and the target protein in the tissue. Since unmodified peptides of GLUT3 were detected via LC-MS/MS, PTM is unlikely to explain the inability of the antibody to detect GLUT3. In addition, SDS-PAGE and Western blotting, as used previously, breaks protein-protein interactions yet was unable to detect GLUT3. This suggests that the GLUT3 antibody epitope is not masked by protein binding. Finally, and most likely is that the antibody may not bind with enough affinity to bovine GLUT3 since there may not be enough sequence homology between the murine and bovine sequences (see Supplementary Figure S6). In comparison to GLUT1, which has significant sequence homology between mouse, human and bovine (Supplementary Figure S6A), the GLUT3 sequence appears more varied between species (Supplementary Figure S6B). Nevertheless, while the present study reports GLUT3 is indeed present throughout the bovine lens, GLUT1 appears to be the major isoform and is present in both epithelial and fibre cells. This is in contrast to the rat lens, where GLUT3 was determined to be the major isoform present in fibre cells (Merriman-Smith et al., 1999; Merriman-Smith et al., 2003), yet the current result is consistent with the previous report by Lim et al. (2017). Future experiments that either selectively inhibit these GLUTs, or use compounds that are selectively transported by these isoforms, may be utilised to determine the relative contributions that each isoform makes to the uptake of glucose in the bovine lens epithelium and peripheral fibres. It is also possible that other isoforms of GLUTs or SGLTs are present in the bovine lens; however, previous Western blotting did not find SGLT1 or SGLT2 in the bovine lens tissue (Lim et al., 2017). Hence, the SGLT transporter family were not targeted for detection in the current study.

Once taken up into cells, we found that SIL glucose was able to be consumed by the three main glycolytic pathways in the lens since SIL glucose metabolites from each pathway could be detected by MALDI-IMS (Figure 6). Unfortunately, a higher number of SIL metabolites could not be detected by either MALDI or GC-MS, possibly because of the transient nature of some metabolites and their rapid metabolism to other metabolic intermediates. The failure to detect other metabolites in the IMS data could be due to the MALDI matrix conditions used, which were optimised for the detection of glucose and its immediate metabolites, and may lack the sensitivity to detect other common endogenous metabolites in glycolysis and the citric acid cycle that could be detected by employing other MALDI matrices (Dekker et al., 2015). In addition, further GC-MS analysis could be performed to target additional SIL metabolites for detection to study metabolic flux in the different lens regions.

While the highest signal intensities for SIL glucose were detected predominantly in the metabolically more active outer cortical regions of the lens, weak signals were also present in the lens nucleus, at longer incubation times (Figures 2, 6, 7). Furthermore, more intense signals for the metabolites SIL G6P and SIL sorbitol, relative to SIL glucose, were also detected in the lens nucleus at these longer incubation times. While the observed SIL glucose uptake pattern may represent uptake *via* GLUTs in the lens epithelium and outer cortical fibres, followed by passive diffusion via an intracellular pathway mediated by gap junctions (Goodenough et al., 1980), the appearance of SIL glucose and its metabolites in the nucleus within 20 hrs is too rapid to occur via passive diffusion alone. It has been predicted that the time required for molecules such as glucose to move into the centre of a bovine lens via simple diffusion to be more on the order of several days (Mathias et al., 1997). Thus while the current experiments cannot rule out the possibility that the observed accumulation of SIL glucose and its metabolites in the lens nucleus occurs via an intracellular pathway, the time course is more consistent with a delivery via an extracellular route as has been recently shown for MRI contrast reagents (Vaghefi et al., 2011). In these experiments, MRI contrast agents were used as an extracellular tracer molecule to show that solutes could be delivered to the nucleus faster than is predicted via passive diffusion (Vaghefi and Donaldson, 2018) *via* a pathway that was localised to the lens sutures (Vaghefi et al., 2012). If indeed SIL glucose was also delivered to the lens nucleus via this extracellular sutural pathway, then the GLUTs, shown to be present in the bovine lens nucleus (Figure 4), would then be able to transport it into nuclear lens fibres, where the local metabolism of this SIL glucose would produce the signals for SIL G6P, and SIL sorbitol observed in our MALDI images (Figures 6, 7). Consistent with this notion of local metabolism of glucose, a previous report has confirmed the presence of aldose reductase in the bovine lens nucleus (Liu et al., 1989). However, due to the previously mentioned spatial resolution limitations of the MALDI-IMS technique, it is not possible to confirm that the observed appearance of SIL glucose and its metabolites in the lens nucleus occurs via an extracellular pathway. While higher spatial resolution techniques will be required to directly visualise this movement, experiments designed to inhibit the microcirculation system (Vaghefi and Donaldson, 2018) will show whether this accumulation of SIL glucose and its metabolites is driven by the microcirculation system.

In summary, we have developed and validated an approach to map the delivery, uptake, and metabolism of glucose in the bovine lens. This approach can not only be used in the future to investigate how glucose is delivered to the lens nucleus by incorporating protocols to perturb the microcirculation, but also to determine what effects exposing the lens to hyperglycaemia have on glucose metabolism in the different lens regions. Hence using this combination of spatially resolved MS techniques, we should be able to determine the role played by the lens microcirculation in the delivery, uptake, and metabolism of glucose in the normal and diabetic lens.

## Conclusion

Glucose is first detected entering the lens in the peripheral epithelial and equatorial regions according to MALDI-IMS. GLUT1 and GLUT3 are present in the bovine lens. GLUT1 is the predominant isoform in lens fibres, GLUT3 is the predominant form in the lens epithelium. Glucose is metabolised primarily in the epithelium and outer cortex, with some evidence of metabolism in the lens nucleus. Higher resolution techniques are required to show extracellular metabolites.

## Data Availability

The datasets presented in this study can be found in online repositories. The name of the repository and accession number can be found below: The European Molecular Biology Laboratory’s European Bioinformatics Institute (EMBL-EBI) Proteomics Identification Database (PRIDE), https://www.ebi.ac.uk/pride/, PXD033022.

## Abbreviations

Da, Dalton; FT-ICR, Fourier Transform Ion Cyclotron Resonance; GC-MS, gas chromatography-mass spectrometry; Gel-LC/MS, gel-based proteomics; GLUT, facilitative glucose transporter; IMS, imaging mass spectrometry; LC-MS/MS, Liquid Chromatography Tandem Mass Spectrometry; MALDI, matrix-assisted laser desorption/ionisation; MeOH, Methanol; m/z, mass-to-charge ratio; NEDC, N-(1-naphthyl) ethylenediamine dihydrochloride; PBS, phosphate buffered saline; SIL, stable isotopically-labelled.
