# Supplementary material for: Mapping Glucose Uptake, Transport and Metabolism in the Bovine Lens Cortex
Source: Front Physiol. 2022 May 31;13:901407. doi: 10.3389/fphys.2022.901407 (PMC9194507; doi:10.3389/fphys.2022.901407)
Supplement: Supplementary file 1 [file DataSheet1.docx]

 **Figure S1: GLUT peptides detected in different bovine lens regions.** Green = Epithelium, Underline = Cortex, Italic = Nucleus.


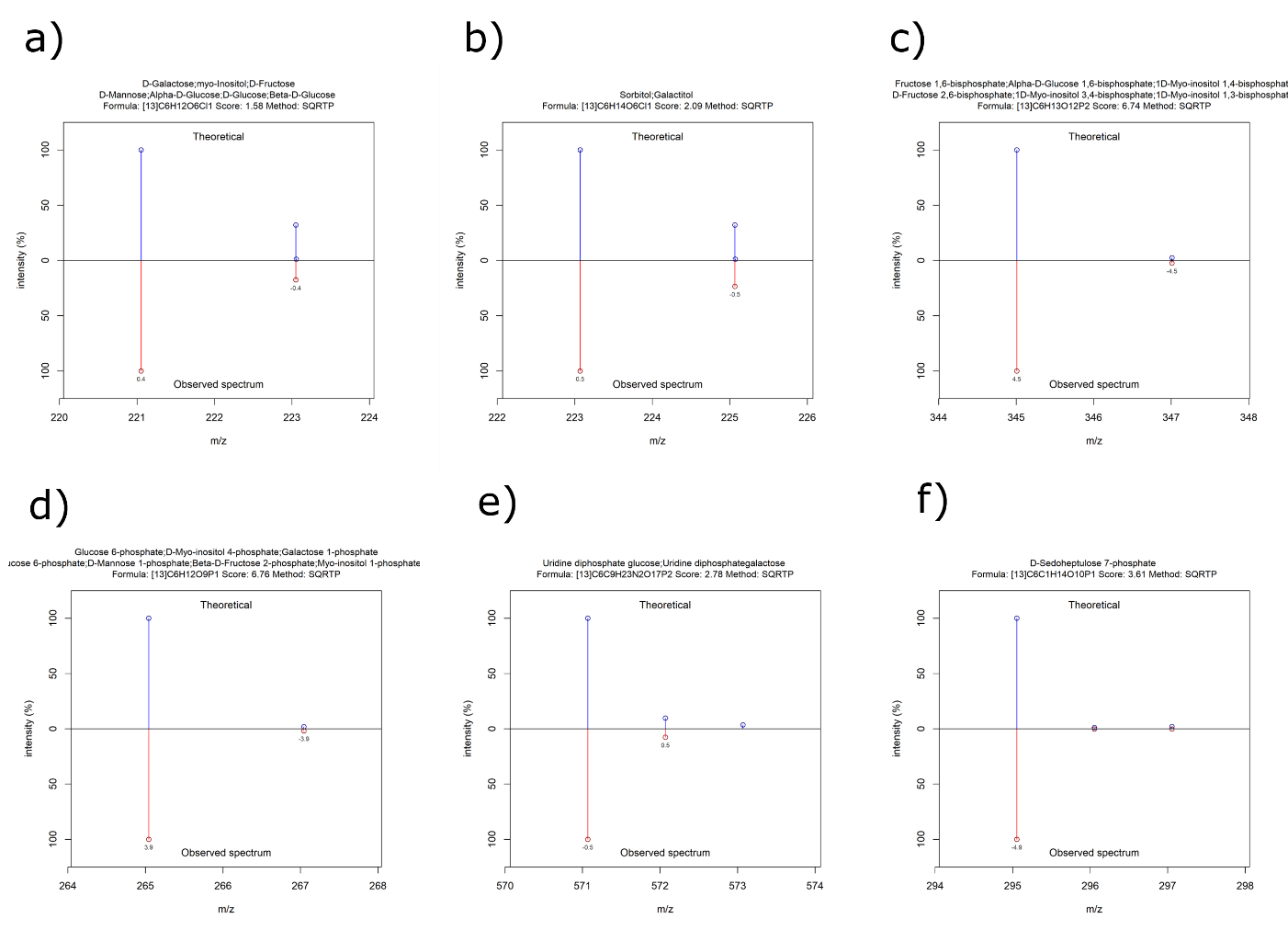


**Figure S2: Isotopic distribution analysis for identification of SIL compounds detected by MALDI-IMS.** The highest scoring spectra (Method: SQRTP) (1) among SIL glucose-incubated samples were selected and reported. A score greater than 1.5 is considered as a high quality spectrum match. (A) SIL glucose [M+Cl]^-^ (B) SIL sorbitol [M+Cl]^-^ (C) SIL fructose-1,6-bisphosphate [M-H]^-^ (D) SIL glucose-6-phosphate [M-H]^-^ (E) SIL UDP-Glucose [M-H]^-^ (F) SIL sedoheptulose-7-phosphate [M-H]^-^ .

1. Guo G, Papanicolaou M, Demarais NJ, Wang Z, Schey KL, Timpson P, et al. Automated annotation and visualisation of high-resolution spatial proteomic mass spectrometry imaging data using HIT-MAP. Nature communications. 2021;12(1):3241.


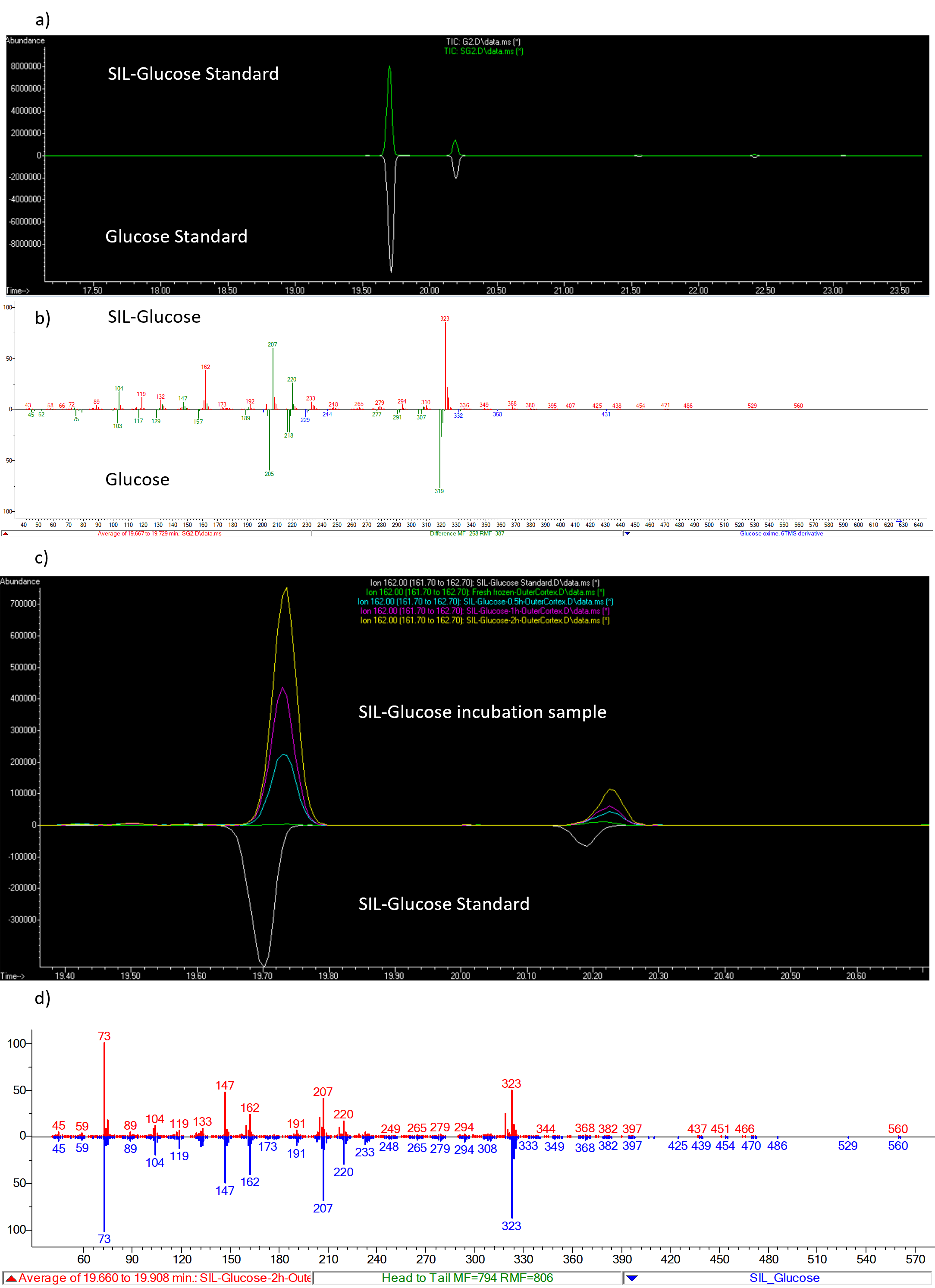


**Figure S3: GC-MS identification of SIL glucose using reference standard.** (A) GC-MS elution profile of standard solutions of SIL glucose (*green*) and glucose (*white*). Two isomeric peaks are present at 19.70 min and 20.20 min. (B) Mass spectrum collected at the retention time 19.70 min (*red*), compared to the reference spectrum for unlabeled glucose extracted from NIST17 database (*blue*). The specific indicative ions of glucose and SIL glucose are highlighted (*green*). (C) The overlaid GC-MS elution profile of SIL glucose incubated lens samples (*top*) and SIL glucose standard (*bottom*). (D) The GC-MS spectrum at 19.70 min from SIL glucose incubated lens samples (*top*) and SIL glucose standard spectrum (*bottom*).


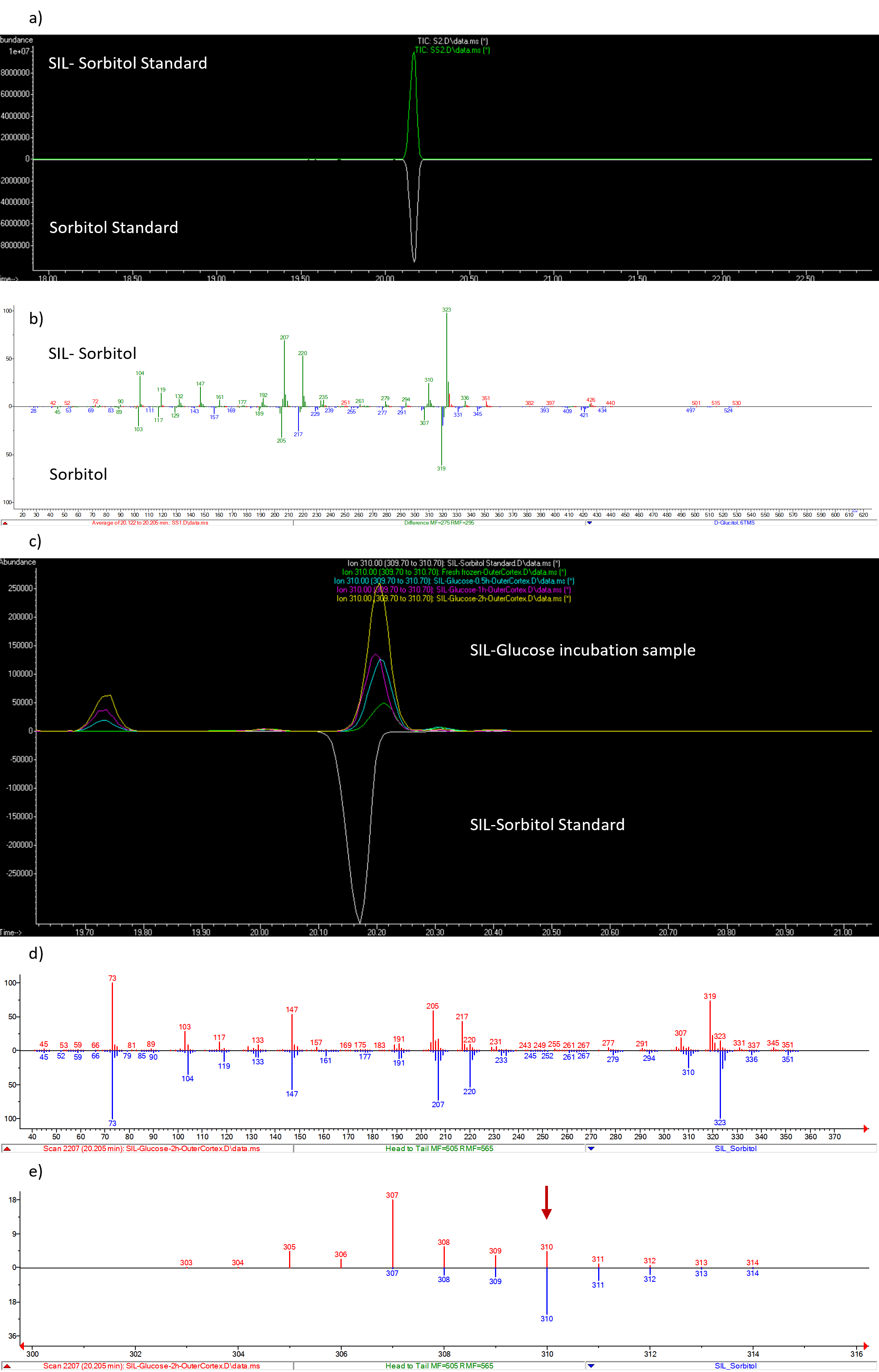


**Figure S4: GC-MS identification of the SIL sorbitol using reference standard.** (A) GC-MS elution profile of standard solutions of SIL sorbitol (*green*) and sorbitol (*white*). (B) Mass spectrum collected at the retention time 20.20 min (*red*), compared to the reference spectrum for unlabeled sorbitol extracted from NIST17 database (*blue*). The specific indicative ions of sorbitol and SIL sorbitol are highlighted (*green*). (C) The overlaid GC-MS elution profile of the SIL sorbitol peak in SIL glucose-incubated samples (*top*) and SIL Sorbitol standard (*bottom*). (D) The GC-MS spectrum at 20.20 min in SIL glucose-incubated samples (*top*) and SIL sorbitol standard spectrum (*bottom*). (E) An enlarged view of selected indicative ion 310 of SIL sorbitol in the spectra comparison window.


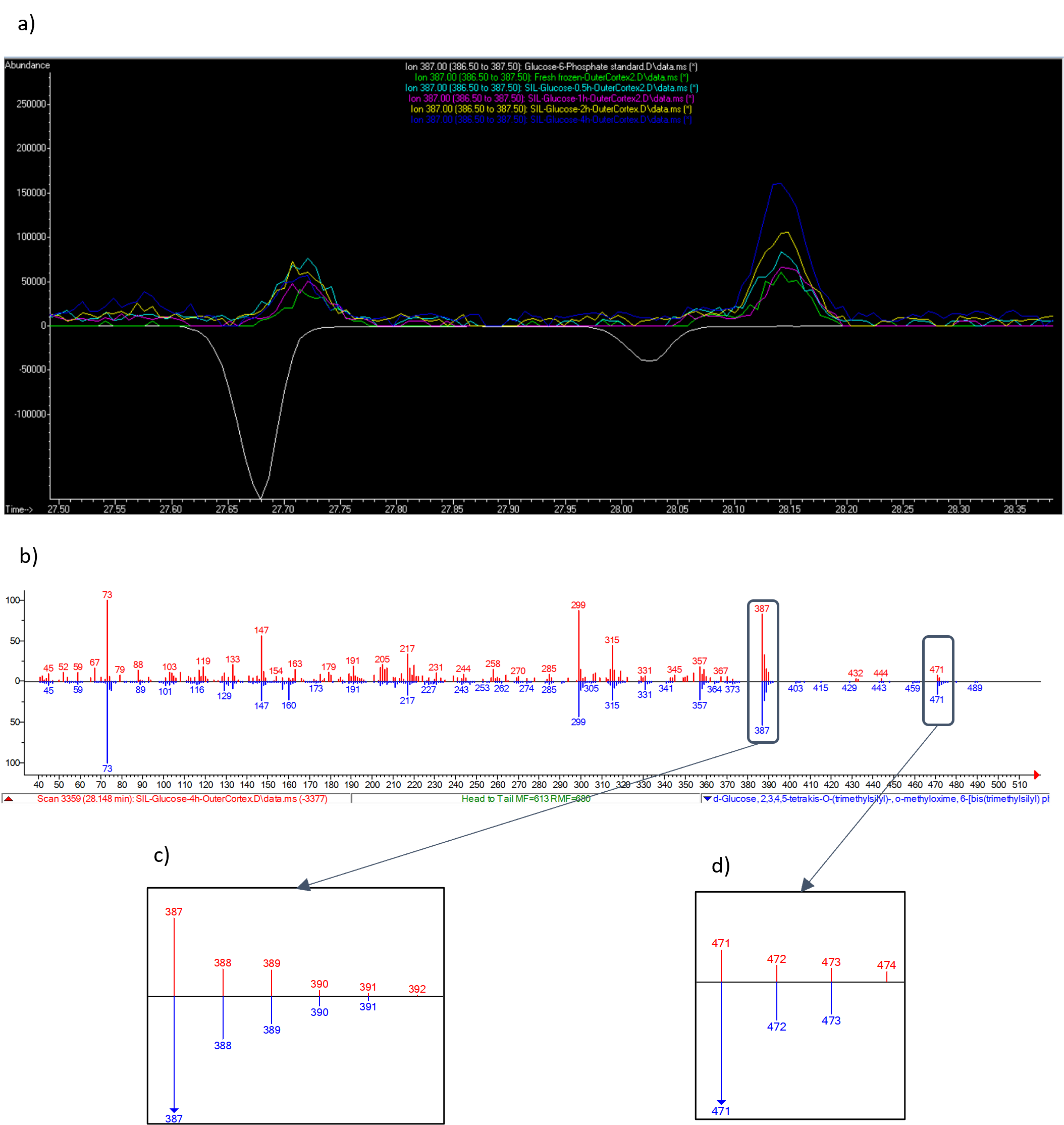


**Figure S5: GC-MS identification of SIL glucose 6-phosphate using reference standard.** (A) Overlaid GC-MS elution profiles of SIL glucose 6-phosphate in SIL glucose-incubated samples (*top*) and glucose 6-phosphate standard (*bottom*). (B) Mass spectra of SIL glucose 6-phosphate in SIL glucose-incubated bovine lens (*red*) and glucose 6-phosphate standard (*blue*). (C) An enlarged view of an ion at *m/z* 387, indicative of SIL glucose 6-phosphate. (D) An enlarged view of an ion at *m/z* 471, indicative of SIL glucose 6-phosphate.

**
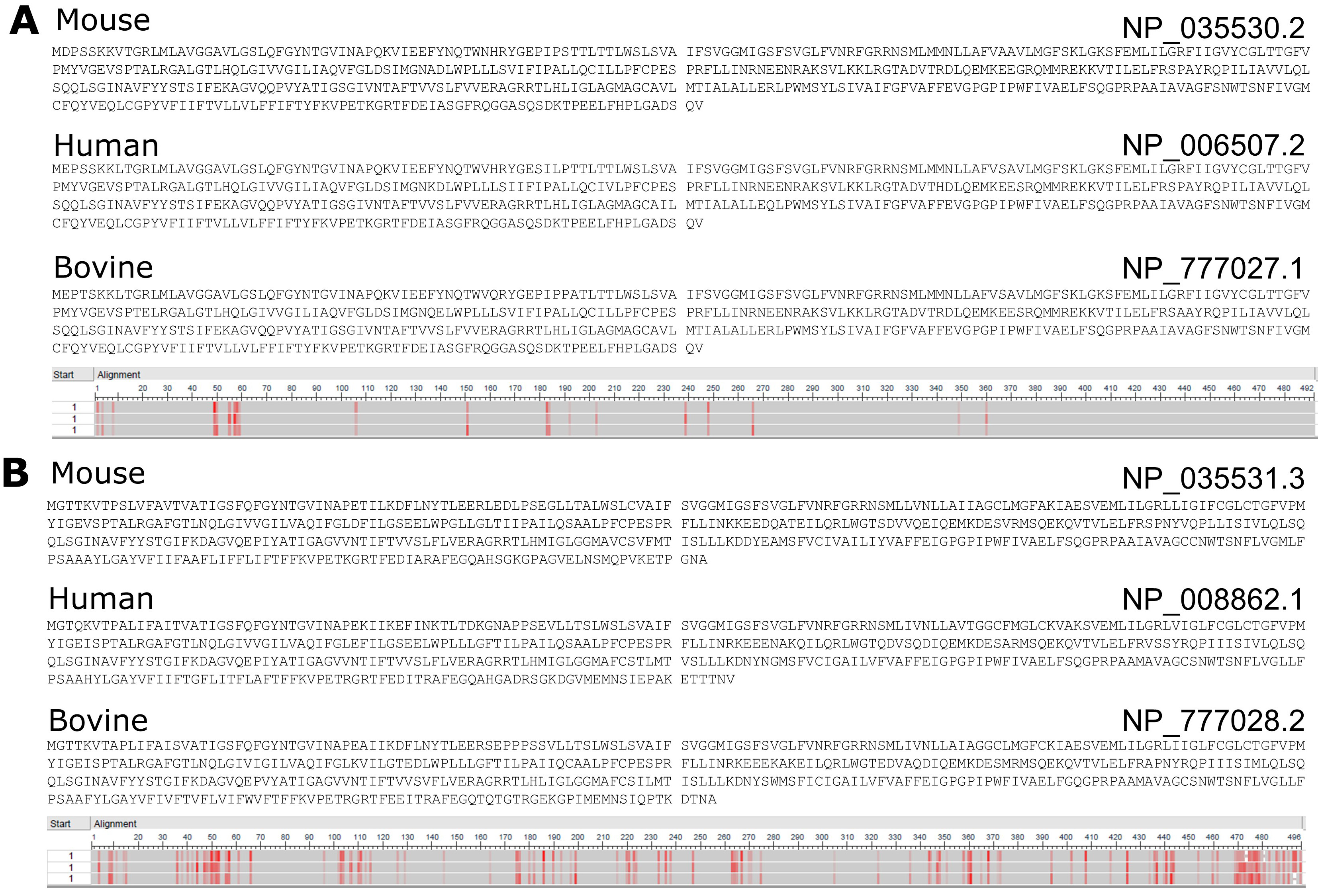
**

**Figure S6: Sequence alignment of (A) GLUT1 and (B) GLUT3.** Areas in red indicate differences in amino acid sequence.

**Table S1: Primary antibodies utilised**

| Antibody Name | Manufacture | Antibody dilution | Epitope Location |
| --- | --- | --- | --- |
| Glucose Transporter GLUT1 peptide (ab202335) | abcam | 1:200 | C-terminus |
| Rabbit Anti-mouse GLUT-1 (GT11) | Alpha Diagnostic International Inc, San, Antonio, TX, USA | 1: 200 | C-terminus, cytoplasmic  domain |
| Rabbit Anti-Mouse GLUT-3 (GT31) | Alpha Diagnostic International Inc, San, Antonio, TX, USA | 1: 200 | C-terminus, cytoplasmic  domain |
| Goat Anti-Mouse GLUT-3 (sc-31838) | Santa Cruz Biotechnology, Inc, Dallas, TX, USA | 1: 200 | N-terminus, extracellular  domain |
| Rabbit Anti-Human GLUT-3 (GT32) | Alpha Diagnostic International Inc, San, Antonio, TX, USA | 1: 200 | C-terminus, cytoplasmic  domain |
| Rabbit Anti-Human GLUT-3 (GT32) | Alpha Diagnostic International Inc, San, Antonio, TX, USA | 1: 200 | N-terminus, extracellular  domain |
